# Removal of Impurities from EAFD Ammonium Carbonate Leachate and Upgrading the Purity of Prepared ZnO

**DOI:** 10.3390/ma16145004

**Published:** 2023-07-14

**Authors:** Zita Takacova, Jana Piroskova, Andrea Miskufova, Tomas Vindt, Maria Hezelova, Dusan Orac

**Affiliations:** Institute of Recycling Technologies, Faculty of Materials, Metallurgy and Recycling, Technical University of Kosice, Letna 1/9, 04200 Kosice, Slovakia; jana.piroskova@tuke.sk (J.P.); andrea.miskufova@tuke.sk (A.M.); tomas.vindt@tuke.sk (T.V.); maria.hezelova@tuke.sk (M.H.); dusan.orac@tuke.sk (D.O.)

**Keywords:** electric arc furnace dust (EAFD), hydrometallurgy, cementation, zinc, iron, lead

## Abstract

The paper describes cementation as a suitable method applied in the refining of EAFD leachates in order to obtain required purity of ZnO for specific industrial application. For study of cementation conditions, the leachate from alkaline leaching with (NH_4_)_2_CO_3_ was used. The leachates contained a high amount of zinc (8000–12,000 µg/mL) and a low content of impurities such as iron, lead, copper, chromium and manganese in the range of 1–21 µg/mL. Cementation conditions were predicted by thermodynamic study, theoretically confirming the viability of the proposed experiments at the considered pH = 8–9. Cementation experiments were carried out using powdered zinc and aluminium (5 g/L) as cementation agents in the first phase separately. To increase the cementation efficiency, their combination was used (2.5:2.5 g/L or 5:5 g/L) at temperatures of 20, 40, 60 and 80 °C for 30 min. The optimum cementation conditions were established as: Zn:Al = 5:5 g/L, 60 °C. Under the given conditions, 100% of Fe, Cu, Pb and Mn were removed from the leachate. The ZnO with the maximum purity of 96.67% was obtained by crystallization of cemented leachate at 105 °C, followed by calcination at 900 °C for 4 h. ZnO with such a purity is suitable for use in the electrical or rubber industries.

## 1. Introduction

Currently, the recycling of electric arc furnace dust (EAFD) is a widely discussed topic, because of its high production (world production of EAFD in 2020 = 37 million tonnes [1]), its hazardous nature and its economic value due to the zinc content. Nowadays, EAFD with a low Zn content (around 3%) is mixed into the pellet/briquette for iron production. For EAFD with a higher Zn content (25–46% [2]), pyrometallurgical processing is preferred, mainly the Waelz Kiln Process (WKP). Almost 90% of produced EAFD is processed by WKP. Crude ZnO as a product of the WKP is subsequently refined by sulphate electrowinning or by the Imperial Smelting Process [3,4]. Hydrometallurgical processing of EAFD is rarely used in industry. However, on a research scale, there are a number of studies describing acidic leaching of EAFD using H_2_SO_4_ [5,6,7,8,9,10,11,12,13], HCl [2,14,15,16], HNO_3_ [2,17,18,19] and other leaching agents [20,21,22]. Leaching with NH_4_Cl is also an option [23].

The problem in acidic leaching of zinc from EAFD is mainly the co-leaching of iron (from ferrite and other Fe oxides), calcium and miscellaneous contaminants such as Al, SiO_2_ and others [5,11,24,25]. Therefore, the obtained leachates should be refined. Precipitation, cementation, ion exchange and other available methods can be used for impurities removal from acidic leachates [25]. Subsequently, zinc can be extracted from the leachate by precipitation in the form of hydroxide or carbonate, or by combination of solvent extraction and electrowinning [2,5,6,8,11,18,21]. Prior to leaching, vortex layer apparatuses [26] can be successfully used to separate the iron phases from the EAFD.

In addition to acidic leaching, alkaline media such as (NH_4_)_2_CO_3_ and NaOH can be used in EAFD processing. The advantage of alkaline leaching agents is the mutual separation of iron and zinc. In alkaline leaching agents, the zinc passes into the leachate and the majority of the iron is concentrated in the insoluble residue. This is mainly because zinc is extracted from ZnO, while zinc ferrite (ZnFe_2_O_4_) is not leached [4,10,18,27,28,29]. Unfortunately, in addition to zinc, a number of accompanying metals are also extracted into the leachate. For example, the most common and frequently identified impurities in EAFD alkaline leachates are Fe, Pb, Cu, Cr, Mn, Cd, Sn, Al, Ni and Si. Potentially Na, K, Ca, etc. may also be present [30,31,32,33,34]. As with acidic leachates, alkaline zinc leachates need to be refined. The reason is to ensure a suitable purity of the leachate for Zn electrowinning and the high purity of Zn compounds for further applications, for instance ZnO. The production of pure ZnO from EAFD has a high potential for industry. The largest consumers of ZnO are the rubber industry (nearly 45%) and ceramics (about 15%) [35]. Possible methods for alkaline leachate refining prior to subsequent precipitation or zinc electrowinning include cementation, sorption, solvent extraction or membrane processes. Cementation is the most common and available option, using zinc as a cementation agent, which also enriches the leachate.

Several studies [5,6,7,8,11,16,21] investigate the cementation of the accompanying metals, but the input is the acidic leachates of EAFD. In contrast, cementation as a refining method for alkaline leachates has not been sufficiently investigated so far, or only marginally so, and detailed cementation conditions are not known. The refining of alkaline leachates by cementation with zinc and the recovery of zinc in the form of ZnO were partially described in [36]. EAFD was leached in (NH_4_)_2_CO_3_ after washing with water. Removal of the present contaminants (Cu, Cd and Pb) was carried out by cementation with zinc dust (5 g/L) for 10 min at 20 °C. After crystallization and calcination, high purity ZnO (99.5%) was obtained. The cementation efficiencies for each contaminant were: Pb—97.1%; Cd—97.3% and Cu—97.1%. In the study of [31], the cementation of Pb and Mn from alkaline leachate of EAFD with zinc was carried, but the other impurities were not discussed. The optimum conditions for reducing the content of impurities to an acceptable concentration are given by the authors as: addition of 2 g/L Zn dust, cementation time = 1 h, temperature = 35 °C and pH of the ammonia solution = 9.03.

The aim of this work was to study the conditions and optimization of Fe, Pb, Cu, Cr, Mn cementation and the way of removal of other present metals (mainly Ca) from ammonium carbonate leachates formed during EAFD leaching and final recovery of ZnO. Based on preliminary experiments, powdered zinc and aluminium were selected as suitable cementation agents, first separately and subsequently in combination. The advantage of using these cementators is their availability and relatively low costs. In addition, zinc is the main metal of interest in the leachate and, by using zinc as a cementing agent, its concentration in the leachate will increase.

The goal was to observe the behaviour of the impurities during cementation at different temperatures and to verify the possibilities and limits for achieving a suitable purity of Zn solution before the subsequent crystallization of the Zn precursor. The ultimate goal was to obtain pure ZnO with suitable properties for its use, for example, in the ceramic, electrical or rubber industries.

## 2. Thermodynamic Study of Cementation of Impurities by Zinc and Aluminium

For the study of thermodynamics, four main contaminants—Fe, Pb, Cu and Cr were chosen. The probable chemical reactions of cementation of these contaminants from zinc ammonium carbonate leachate using two cementation agents—Zn and Al at two considered temperatures (20 and 80 °C) were calculated using HSC Chemistry v6.1 software [37], Table 1 and Table 2. Temperatures of 20 and 80 °C were chosen as the two boundary temperatures to be considered for the cementation experiments, to show the tendency of the system behaviour with increasing temperature.

**Figure 1:**
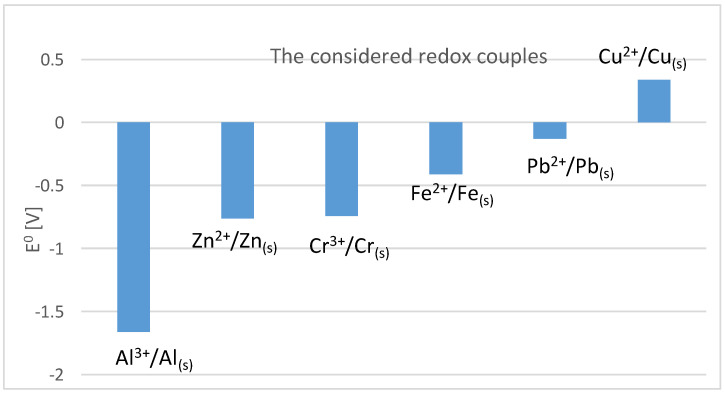
E° values of redox pairs of the present metals [38].

On the basis of the calculated ΔG^0^ values, the cementation order of the individual metals was determined using zinc and aluminium cementators as follows: Cu → Pb → Fe → Cr. This order is consistent with the values of standard electrode redox potential pairs, Figure 1.

**Figure 2:**
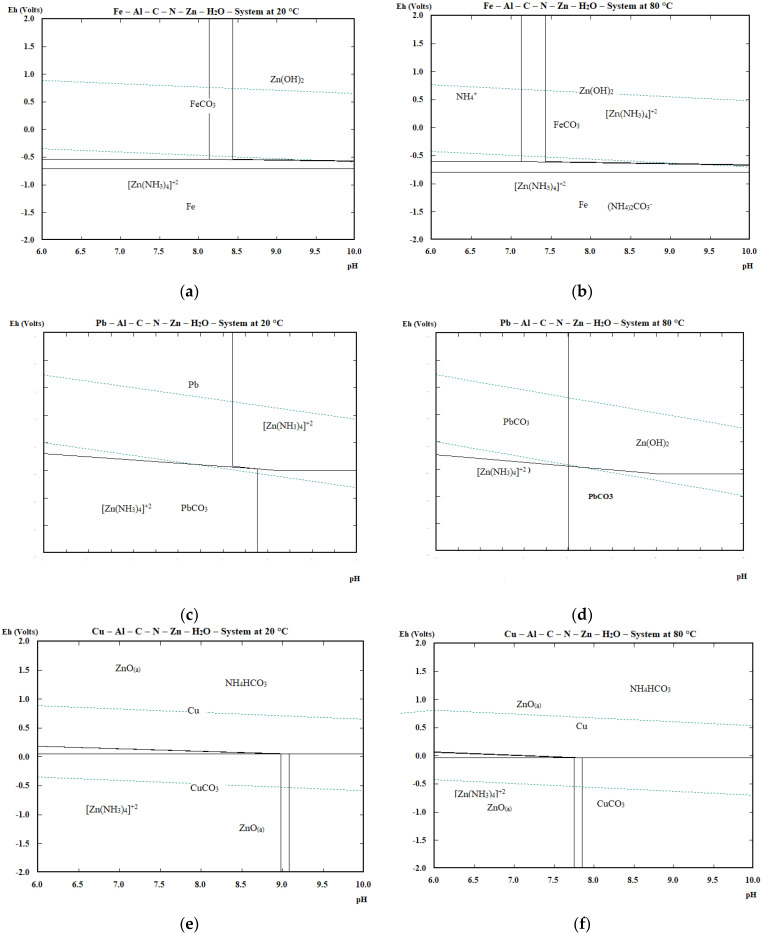

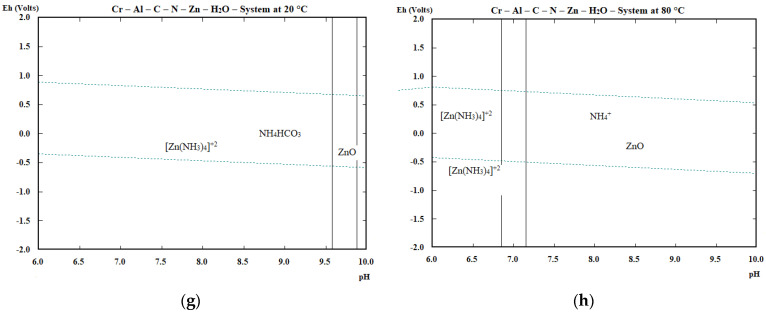
E–pH diagrams for Me–Zn–Al–C–N–H_2_O system at 20 and 80 °C at atmospheric pressure, where Me = Fe (**a**,**b**), Pb (**c**,**d**), Cu (**e**,**f**), Cr (**g**,**h**) with molality of 1 × 10^−4^ mol/kg for Fe, Pb, Cu and 8 × 10^−5^ mol/kg for Cr.

In order to predict the behaviour of the impurities in the leachate during cementation, E–pH diagrams for the Me–Zn–Al–C–N systems (where Me = Fe, Pb, Cu, Cr) were constructed with the HSC Chemistry 6.1 [37] at both considered temperatures (20 and 80 °C), Figure 2a–h.

For the construction of the diagrams, a pH range of 6–10 was selected and the presence of the solid phases of the examined metals was evaluated at pH = 8–9, which represents the real pH of the input leachate. At the same time, the maximum concentrations of the cemented metals in the leachates were also taken into account (expressed in mol/kg).

Stability region of metallic iron in the presence of (NH_4_)_2_CO_3_ and other dissolved impurities is located in the areas outside the stability of the water over the whole considered pH range, therefore it can be expected that, in the presence of zinc and aluminium at the considered pH = 8–9, iron can be removed from solution as FeCO_3_ rather than as metallic at both considered temperatures. On the other hand, Fe is removed by Zn and Al from the investigated metals with a lower probability from a thermodynamic point of view. Higher temperatures only slightly extend the region of existence of elemental Fe in the alkaline pH beyond the water stability limit. Lead exists at pH = 8–9 as Pb and PbCO_3_ at 20 °C. At 80 °C there is no area of metallic Pb and it could probably be removed from solution as carbonate. Copper can be removed out of solution as Cu or CuCO_3_ at pH = 8–9 at both studied temperatures. In the case of chromium, there is no phase in E–pH that would predict the cementation of chromium, e.g., in metallic form, although the cementation reactions are thermodynamically viable.

The thermodynamic study confirmed the probability of the proposed cementation reactions of accompanying metals such as Fe, Cu, Pb and Cr using powdered zinc and aluminium, where ΔG^0^ of most of the expected reactions takes a negative value at both considered temperatures, and hence should proceed in the direction of product formation. The E–pH diagrams confirmed the presence of the solid phases of the studied metals at the considered pH = 8–9, with the exception of chromium. At 80 °C, the cementation process may differ from 20 °C, also due to the decomposition of (NH_4_)_2_CO_3_ and the presence of other nitrogen, carbon and water based species and the change in the activity of the individual species, since at higher temperatures (ca. 50 °C) the decomposition of (NH_4_)_2_CO_3_ and the release of gaseous NH_3_ and CO_2_ occur according to the Reaction (19)

(NH_4_)_2_CO_3_ = 2NH_3(g)_ + CO_2(g)_ + H_2_O, ΔG^0^_353_ = −6.2 kJ (19)

**Figure 3:**
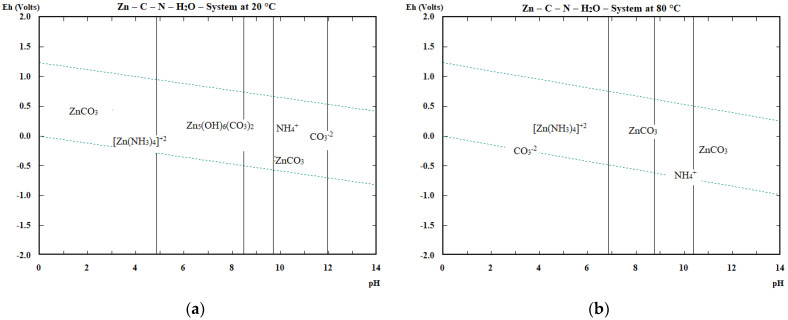
Passivation of zinc in the Zn–(NH_4_)_2_CO_3_ system (**a**) at 20 °C (**b**) at 80 °C.

**Figure 4:**
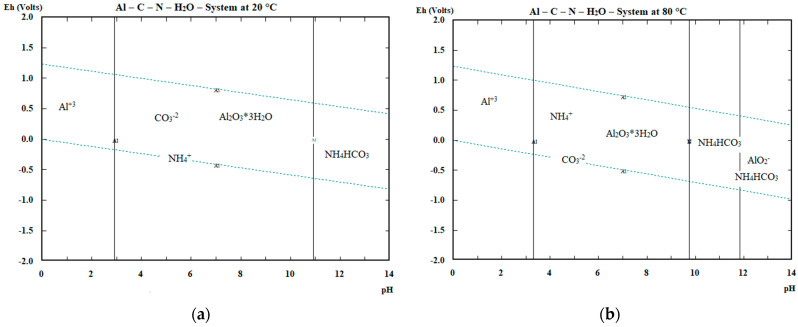
Passivation of aluminium in the Al–(NH_4_)_2_CO_3_ system (**a**) at 20 °C, (**b**) at 80 °C.

The question on a practical scale is also the passivation of powdered Zn and Al and its influence on the reactions proceeding, form of cementation products and their removal rate under specific conditions and finally the practical efficiency of accompanying metals’ cementation. The passivation of the Zn and Al is illustrated via E–pH diagrams at 20 and 80 °C in Figure 3 and Figure 4.

The passivation of zinc in the cementation process at pH = 8–9 seems to take place by ZnCO_3_ formation mainly at 20 °C. At higher temperatures, Zn_5_(OH)_6_(CO_3_)_2_ exists in addition to ZnCO_3_. In case of aluminium the passivation occurs mostly through the formation of hydrated alumina at 20 °C in the considered region of pH = 8–9, up to pH~11. At 80 °C, the hydrated alumina presence range is reduced to below pH 10. The presence of alumina and hydrated alumina is also predicted by the cementation reactions given in Table 2. In case of using both cementing agents at the same time, the formation of a complex compound based on ZnO.Al_2_O_3_ is more probable (Reaction (20)) than individual Zn and Al oxides/hydroxides/carbonates from the thermodynamic point of view.

2Al + Zn + 4H_2_O + 8CO_3_^−2^ = ZnO*Al_2_O_3_ + 8HCO_3_^−^ + 8e^−^, ΔG^0^ _353_= −1555 kJ (20)

## 3. Materials and Methods

For the experimental study of cementation, the leachates produced by leaching the EAFD in ammonium carbonate were used. Previously, a neutral leaching of the EAFD was carried out to remove the chlorides present.

For cementation experiments powdered zinc and aluminium were used, both in analytical grade p.a. (Centralchem, Bratislava, Slovakia).

The chemical analysis of EAFD, leachates and obtained product were carried out by the AAS method using Varian AA240+ or Thermo Scientific (iCE 3000 series, London, UK) with the stock solutions at a concentration of 1 g/L ± 0.002 or 0.005 for metals such as Fe, Cu, Pb, Cr, Al, Si and others, and at a concentration of 10 g/L ± 0.020 for zinc. The results from the AAS analysis are acceptable for this method with a relative standard deviation value below 6% for three replicate measurements. By AAS, the content of Zn, Fe, Pb, Cd, Cu, Cr, Mn, Ca, Si and as well Al in the case of cementation with Al was determined. Chloride content was determined by titration and sulphate by a UV/VIS spectrophotometer HI83099 Hanna Instruments (Smithfield, RI, USA).

The chemical composition of the input EAFD and the dust after neutral leaching is given in Table 3. Mineralogical study of EAFD showed that phases such as franklinite ZnFe_2_O_4_, zincite ZnO, magnetite Fe_3_O_4_, limestone CaCO_3_ and silica SiO_2_ are predominantly present in the sample. 

Analysis of the tap water intended for neutral leaching from an external source is given in Table 4. 

The chemical composition of the leachates from the ammonium carbonate leaching of the EAFD as an input for the cementation experiments is given in Table 5. Leachate 1 was obtained by a semi-operating experiment, (S/L = 10, 100 g/L (NH_4_)_2_CO_3_), 50 °C, 30 min). Leachates 2 and 3 were obtained by repeated leaching the washed EAFD in the initial leachate with the addition of 25 g/L (NH_4_)_2_CO_3_ (analytical grade, p.a., microCHEM, Pezinok, Slovakia) for 30 min, S/L = 10 and 50 °C.

Cementation of enriched leachates (leachates 2 and 3) was carried out in a glass laboratory reactor, which was placed in a thermostatically controlled water bath. The input volume of the cemented leachates was 300 mL. The pH of the leachates was measured using a WTW inoLab pH meter (pH/ION7320, Burladingen, Germany) before and after the cementation experiments. Cementation with zinc (5 g/L) or aluminium (5 g/L) separately was carried out under the following conditions: 20 and 60 °C, 30 min, 300 rpm. 

To study the effect of the combination of cementation agents (Zn:Al = 2.5:2.5 g/L or 5:5 g/L) on the process efficiency, the experiments were carried out at 20, 40, 60 and 80 °C, 30 min at 300 rpm. Preliminary experiments were also carried out at 20 °C with the combination Zn:Al = 7.5:7.5 g/L, but without a significant increase in cementation efficiency.

The cementation efficiency was calculated according (21)

(21) $$\mu =\frac{c_{\left(0\right)}-c_{\left(1\right)}}{c_{\left(0\right)}}\cdot 100\;\left(\%\right)$$

where c_(0)_ is the initial metal concentration in the leachate and c_(1)_ is the metal concentration in the leachate after cementation. The resulting efficiencies represent the average value obtained from three measurements (three samples taken and analysed after cementation).

The solid residues after washing and drying (100 °C, 24 h) were analysed by XRD using a Philips X’Pert PRO MRD (Co-Kα), range of measuring (10–120° 2theta), scan step (0.0170°) diffractometer (Philips, Amsterdam, The Netherlands). The phases were identified using X’Per HighScore plus software, v3.0a (3.0.1). Selected samples were subjected to XRF (Shimadzu EDX 7000 (Tokyo, Japan) and scanning electron microscopy (SEM) analysis along with energy-dispersive spectrometry, MIRA3 FE-SEM (resolution: 1.2 nm at 30 kV; 2.3 nm at 3 kV, TESCAN, Warrendale, PA, USA). Selected cementation residues were subjected to X-ray photoelectron spectroscopy (XPS) using a Kratos AXIS ULTRA DLD spectrometer (Kratos Analytical, Manchester, UK) with: X ray monochromatic Al Kα (1486.6 eV), 150 W, charge neutraliser system, analysis area: 300 × 700 µm^2^_,_ energy calibration C 1s = 284.7 Ev.

The leachates after cementation were further subjected to crystallization and calcination in order to obtain pure zinc oxide. Crystallization was carried out at 105 °C. The crystallization products were subjected to calcination at 900 °C for 4 h. The calcinates were analysed for the content of the zinc and accompanying metals by AAS. In addition, SEM—EDX analysis and XRD were also carried out on selected ZnO samples.

## 4. Results and Discussion

### 4.1. Metal Cementation from Ammonium Carbonate Leachate

The results of cementation of the accompanying metals using zinc are shown in Table 6 and using aluminium in Table 7.

The results of cementation with the combination of Zn:Al = 2.5:2.5 g/L and 5:5 g/L at 20, 40, 60 and 80 °C are given in Table 8.

The achieved results of cementation with Zn (Table 6) show that, even at 20 °C, almost complete removal of Cu, Cr and Mn occurred, but only 3% of Fe was removed. Increasing the temperature had a positive effect on the cementation of iron, because, at 60 °C, 100% Fe cementation efficiency was achieved. In the case of Pb, only partial removal occurred at both used temperatures (20 °C—48.3%, 60 °C—70%). When aluminium was used as cementation agent (Table 7), about 25% of Fe was cemented at 20 °C. A better but not completely satisfactory result was obtained at 60 °C—61%. Cr and Mn cemented with 100% efficiency already at 20 °C. Cu reached a maximum cementation of 90% at 60 °C, Pb was cemented in the range of 64–68%. At the same time, almost 70% of the calcium was removed in the process when both cementators had been used at both temperatures.

The results correlate with the thermodynamic study. In the case of Cr and Mn, it is shown that, in a given leaching medium, it does not depend on the type of cementation agent and complete cementation occurs under the given conditions.

The insufficient cementation efficiencies of Fe, Pb and partially of Cu resulted in experiments that combined both cementation agents (Table 8). Two leachates were applied for cementation, namely leachate N° 2 at 20 and 60 °C and leachate N° 3 at 40 and 80 °C (the chemical composition of these leachates is in Table 5).

In addition to the elements listed in Table 8, the Cd, Si and Al contents were also determined but were under the detection limit of the AAS method. In all leachates where aluminium was used as a cementation agent there was no transfer of aluminium to the leachate. probably indicating its significant passivation in the form of insoluble Al_2_O_3_ or its precipitation from the leachate as Al(OH)_3_. Aluminium may act as an electron donor with subsequent precipitation from the leachate [40,41]. However, its absence in the leachates can be considered as a large advantage from the point of view of their further processing.

The observed cementation results confirm the significant effect of temperature in the cementation of iron. While at 20 °C iron cementation efficiency of 38.8% was achieved using Zn:Al = 2.5:2.5 g/L, at 60 °C, the efficiency increased to almost 80% on otherwise identical terms.

At 80 °C, complete removal of iron from the leachate was achieved even at lower weights of cementators. The cementation of Pb and Cu is not significantly affected by temperature; at 20 °C maximum removal efficiency (100%) of both metals was achieved. For chromium cementation, the efficiency even decreases with temperature in some experiments. In addition to the main impurities, the calcium content of the leachates after cementation also decreased. The highest efficiency of calcium removal was obtained at 80 °C at Zn:Al = 5:5 g/L.

A temperature of 60 °C can be considered sufficient to remove the majority of the impurities, where 100% removal efficiency of Fe, Pb, Cu and 57% removal efficiency of Cr can be achieved using a combination of Zn:Al = 5:5 g/L. A lower proportion of cementators leads to a reduction in the cementation efficiency of Fe under otherwise identical conditions, but Pb and Cu are removed to 100%.

Interestingly, while the efficiency of chromium cementation using zinc and aluminium as cementation agents separately was 100%, using their combination it dropped to a maximum of 57%. The reasons for the preferential cementation and removal of Fe, Cu and Pb over Cr are probably due to the change in the redox potential value of Al−Zn pair in ammonium carbonate solution due to competitive interaction of those cementation agents and at the same time the change of the overall redox potential of Al−Zn−Me system (Me = accompanying metal ion in ammonium carbonate solution) after reaching the steady state (quasi-equilibrium under given conditions) in comparison to the Al−Me system and Zn−Me system and extending the cementation time >30 min could lead to complete removal of chromium.

### 4.2. Characterization of Cementation Residue

The aim of XRD qualitative phase analysis of cementators under different conditions was to describe or indicate the probable presence of cementation products or the possible formation of by-products during cementation. However, due to the low concentrations of metals in solution, the results of the XRD qualitative phase analysis should be taken as a guideline.

In the case of the use of pure Al (5 g/L) as the cementator at 60 °C, the possible presence of Fe and Cr in elemental form was identified, while the presence of Pb and Ca was not confirmed by the given measurement. For Ca, due to its electronegativity, its removal in elemental form was not expected. However, due to the decrease in its concentration during cementation, it was assumed that Ca could be removed as solid CaCO_3_, but this was not confirmed under the given conditions.

Copper was identified as CuCl and CuO and manganese as Mn_3_Si. Moreover, manganese has the thermodynamic ability to bind with the present silicon (either in solution or as an impurity in the Al cementator). Zinc from the carbonate solution reacted with the Al cementator to form the most likely compound ZnAl_2_O_4_, which is consistent with the thermodynamic prediction in the Zn-Al-H_2_O (or (NH_4_)_2_CO_3_) system.

The aluminium remained mostly in the elemental form after cementation at 60 °C (as well as at 20 °C) and no other corrosion products except ZnAl_2_O_4_ based on aluminium like hydroxides or oxides could be identified. The cemented metals were mostly subject to oxidation and reactions with the cementation matrix as well as other present impurities. This depended on the actual process conditions and the affinity of the metal ions in solution for the given concentration and temperature.

In the case of zinc cementation at 60 °C, the presence of a ZnO corrosion product is evident, which also agrees with the thermodynamic calculation. At the same time, the presence of hydrozincite Zn_5_(OH)_6_(CO_3_)_2_ and Zn_3_Cu_2_(OH)_6_(CO_3_)_2_, respectively, was indicated. Copper, in addition to Cu-hydrozincite, can be bound as CuO or CaCuO_2_. Iron could be present as Ca_2_Fe_2_O_5_ rather than the elemental Fe and manganese as Mn_2_O_3_. Calcium was identified as CaO and Ca(OH)_2_ in addition to the above mentioned compounds. Lead could also be present as CaPb_3_.

**Figure 5:**
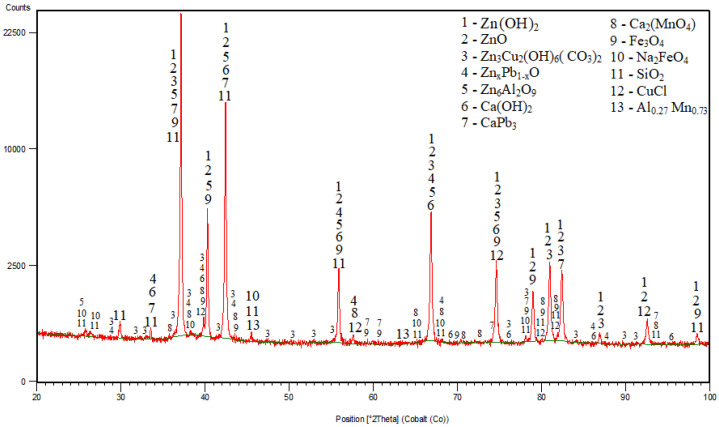
Cementation residue from experiment N° 10 (Zn:Al = 5:5 g/L, 60 °C).

In the case of a mixture cementators, the situation is slightly different. The XRD pattern of the cementation residue from experiment N° 10 where the mixture Zn and Al was used (Zn:Al = 5:5 g/L, 60 °C), is shown in Figure 5.

After 30 min of cementation, practically no elemental aluminium or zinc was identified. XRD analysis indicates an almost complete surface reaction of aluminium with zinc and their oxidation in the studied leaching system during cementation experiment to form the compound 6ZnO.Al_2_O_3_. Zinc was identified mainly as ZnO and Zn(OH)_2_. Iron was identified as Fe_3_O_4_ and Na_2_FeO_4_. Copper was possibly present as CuCl and Cu-hydrozincite and lead as Zn_x_Pb_1-x_O and CaPb_3_. Moreover, calcium was identified as Ca(OH)_2_ similarly to the pure Zn cementators at 60 °C and together with manganese as Ca_2_MnO_4_. In addition to the investigated metals, minor concomitant elements such as chlorine, sodium and potassium (e.g., identified in CuCl, Na_2_FeO_4_) may also be present in the cementation residue.

From Figure 5 it is visible coincidence or overlapping of some diffractions (peaks) which belong to or can be associated with diffractions of more than one individual phase in the diffraction pattern of the whole mixture. Based on the standard qualitative diffraction phase analysis procedure by using the HighScore software, v3.0a (3.0.1), taking into account also that the phase diffractions coincide together with the thermodynamic assumption and probability calculations, the most probable phases have been identified and selected for the given cementation system.

**Figure 6:**
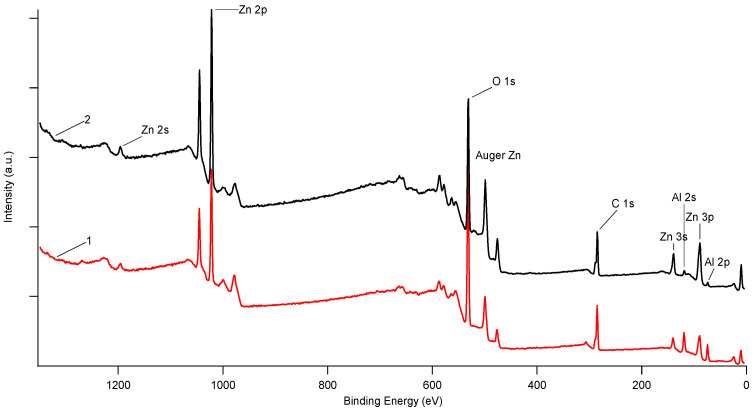
XPS spectra of cementation residues (from experiment N° 4—spectrum 1, from experiment N° 10—spectrum 2).

The complete surface oxidation of Al during cementation with the mixture (Zn:Al = 5:5 g/L, 60 °C) is also confirmed by XPS analysis—spectrum 2 (Figure 6). Spectrum 1 represents the sample after cementation using pure Al (5 g/L, 60 °C) for comparison. The XPS spectra indicated that both samples contained zinc, confirming the participation of zinc ions in the reaction during using pure Al as cementator in carbonate solution and its deposition and further reactions on the Al surface.

The obtained results showed that the combination of the two cementation agents (Zn + Al) is a more complex process and caused more significant oxidation of the cementators as well as cemented impurities on the surface of the cementators. This was in contrast to the use of the cementation agents separately, where the oxidation rate of the cementators on their surface after 30 min was considerably lower and moreover created different Zn phases (ZnO and hydrozincite). On the contrary, in the cementation residue, when both of Zn and Al have been used, zinc is probably preferentially bound with Al as Zn_6_Al_2_O_9_. Subsequently, ZnO and Zn(OH)_2_ are generated, but the formation and ratio of individual Zn phases during cementation will be influenced at least by temperature, overall redox potentials of the solution or potentials of individual metal pairs, content and concentration of dissolved impurities in the carbonate solution.

The cementation process seems to be quite easy to perform but, on the other hand, it is rather complicated from a mechanism point of view due to the complexity of the leaching system. Moreover, the effectivity and kinetics of the cementation will play roles and also other phenomena like reactions on the three phase boundary (liquid, solid, gas), where there are also dissolved gases (e.g., H_2(g)_) due to the reaction of Al and Zn with water and NH_3(g)_ from decomposition of ammonium carbonate. XRD analysis also indicated that calcium is removed from the solution mostly as Ca(OH)_2_ rather than CaCO_3_, which is more probable from a thermodynamic point of view. On the other hand, it was also proved that calcium could be separated from the solution not only as precipitated solid Ca(OH)_2_ but also by reaction with other cemented impurities such as Fe, Mn and Pb which form specific compounds.

### 4.3. Characterization of Obtained Calcinates

The chemical composition of the calcinates obtained by crystallization and subsequent calcination of the refined leachates from experiments N° 1–4 are given in Table 9.

**Figure 7:**
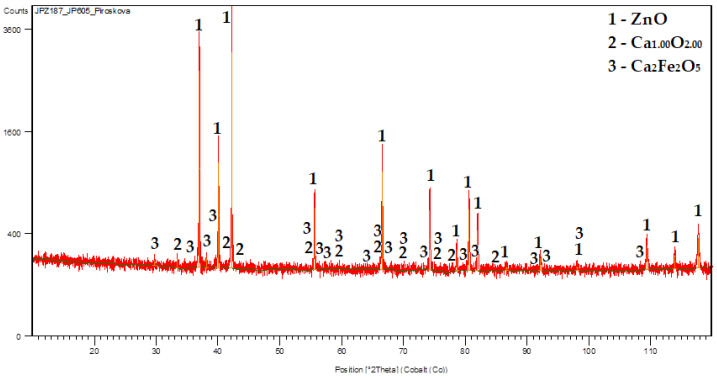
Calcinate (73.13 wt.% of Zn) from experiment N° 1 (5 g/L Zn at 20 °C).

**Figure 8:**
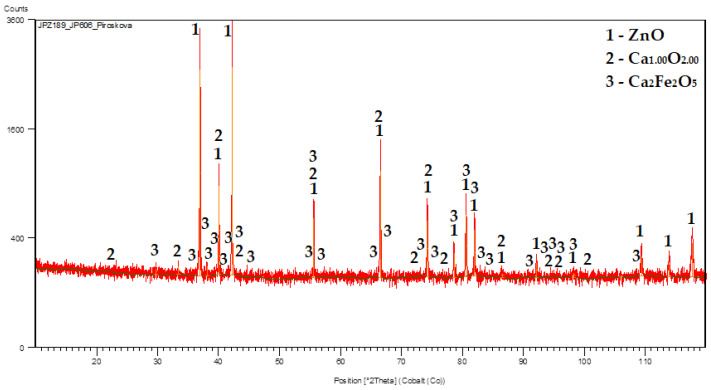
Calcinate (62.74 wt.% of Zn) from experiment N° 3 (5 g/L Al at 20 °C).

The XRD patterns of the selected calcinates are shown in Figure 7 and Figure 8.

The chemical composition of the calcinates obtained by crystallization and subsequent calcination of the refined leachates from cementation experiments N° 5–12 are given in Table 10.

**Figure 9:**
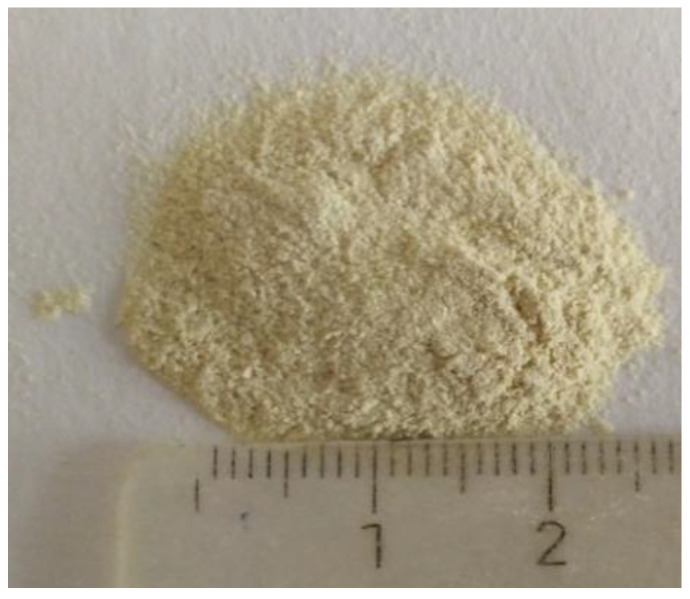
ZnO with the highest Zn content (from Experiment N° 10).

**Figure 10:**
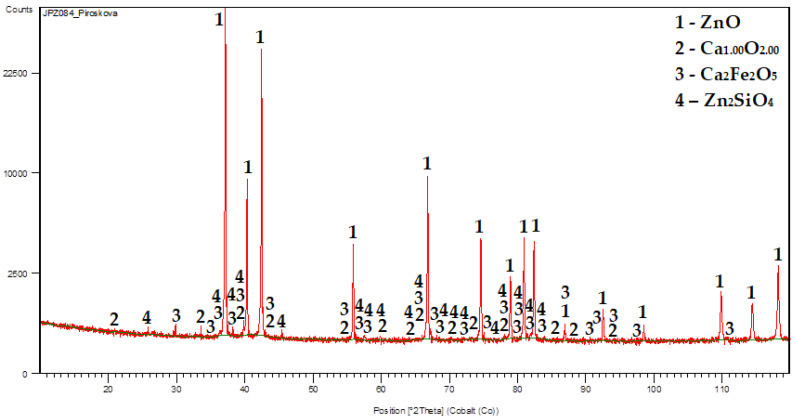
XRD pattern of ZnO with the highest Zn content.

**Figure 11:**
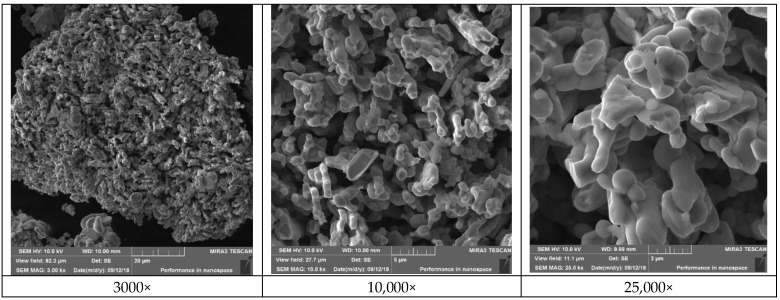
Morphology of ZnO with the highest Zn content.

**Figure 12:**
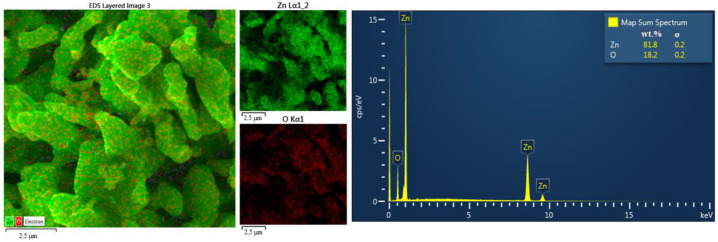
EDX analysis of ZnO with the highest Zn content.

ZnO with the highest zinc content (from Experiment N° 10) is shown in Figure 9. The results of its XRD analysis, SEM at magnifications of 3000×, 10,000× and 25,000× and EDX analysis are shown in Figure 10, Figure 11 and Figure 12.

The Zn, Fe, Pb, Cu, Cr, Mn and Si contents were determined in selected calcinates prepared by crystallization of the refined leachate and subsequent calcination of the obtained product. It was confirmed that, at a cementation efficiency of 100% of accompanying metals such as Cu, Pb and Mn, calcinates did not contain these metals or their content was below the limit of detection.

From the obtained results (Table 9), it can be concluded that the zinc content in the ZnO ranged from 74.47% to 77.67%. The highest zinc content (77.67%), which corresponds to ZnO content of 96.67%, was obtained from Experiment N° 10 (Zn:Al = 5.0:5.0 g/L, 60 °C). From the XRD pattern shown in Figure 10, the majority presence of the ZnO phase can be observed. The minor contaminant is the dicalciumferrite Ca_2_Fe_2_O_5_, which corresponds to the chemical analysis presented in Table 5. The presence of willemite—Zn_2_SiO_4_ was also confirmed, while silica can be considered as a stable contaminant with no significant influence on the quality and applicability of the final product. The ZnO particles (Figure 11) are of the mostly elongated or irregular shape, less of a globular shape with a dimension range mostly from to 5 μm in length and 1 micrometre in diameter for the individual particles which are formed to the bigger clusters of around 100 μm in diameter. The form of the particles indicates the sintering of individual particles due to the high calcination temperature. It can be seen that the EDX mapping analysis confirmed the major content of zinc and oxygen. From the economic point of view, and as further investigations of the research team showed [42], for selected ZnO applications (e.g., tyre production), a lower calcination temperature (around 400–500 °C) can be also considered while maintaining sufficient purity and the required properties, since at 700–900 °C the particles are sintered and the specific surface area is reduced.

**Figure 13:**
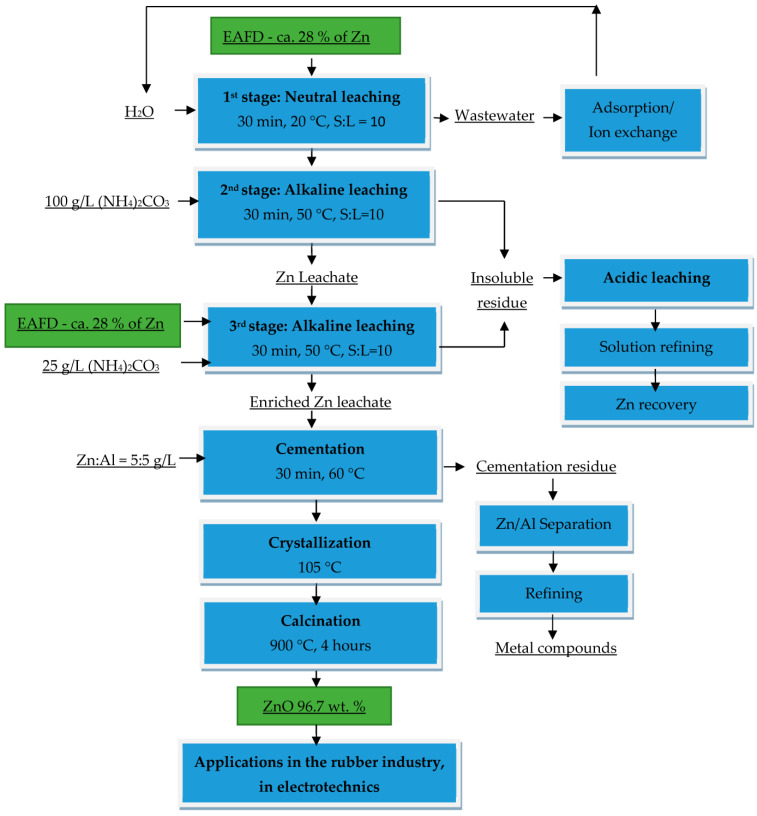
Proposal of comprehensive hydrometallurgical treatment of EAFD with the aim to recover pure ZnO product.

On the basis of the results from neutral and alkaline leaching published previously by us as the authors [43,44,45,46,47], and according to the results of cementation, a comprehensive processing for EAFD was proposed, Figure 13.

The aim is to obtain a high-purity ZnO with minimal impurities of Fe, Ca and Si, which can be used, for example, in the rubber industry. In the literature [48], the minimum purity of ZnO for application in the rubber industry is reported to be 93%, with lead and cadmium contents of 3 and 2 ppm, respectively. The final product of ZnO meets the required purity. The obtained ZnO has particles of irregular elongated shape, while according to [49] the rubber industry prefers a spherical shape of the ZnO particles. The practical application of the obtained ZnO in the rubber industry could be the subject of further investigation as well as the modification of the ZnO particles’ shape by changing the parameters of crystallization and the calcination process of the intermediate Zn product.

According to [50,51], ZnO with such purity can also be used in the production of varistors. The impurities in the form of Si, Ca and Fe do not reduce the quality of the component in terms of electrical properties, moreover, they could even act as a required dopant.

## 5. Conclusions

The efficient treatment of EAFDs is a highly topical issue due to their quantity, hazardous nature and high zinc content as the main metal of interest (about 30 wt.%). In this work, a possible procedure for the refining of alkaline leachate was proposed and verified, which represents one of the steps in the comprehensive processing of the EAFD presented in the scheme, Figure 13. The alkaline leaching process ensures the selective transfer of zinc from the ZnO phase to the leachate without leaching the major amount of iron, the content of which in the leachate is at a similar level to the zinc content (approximately 30 wt.%). The insoluble ZnO-depleted residue is subsequently treated by acidic leaching to obtain iron and residual zinc from the ZnFe_2_O_4_, which represents the second treatment stream.

The zinc-containing leachates obtained by alkaline leaching have to be refined from the accompanying metals, which are iron, lead, copper, chromium, manganese, etc. Cementation is a simple, cheap and reliable method of refining these leachates and, in addition, in the case of zinc as a cementation agent, the leachate is enriched in zinc.

The combination of Zn and Al as cementation agents proved to be the most effective for the experiments carried out, where, under optimum conditions (Zn:Al = 5:5 g/L, 60 °C, 30 min), a substantial part of the contaminants was removed, in some cases with 100% efficiency (Fe, Cu, Pb and Mn). At the same time, aluminium was not transferred to the leachate. In contrast to cementation with zinc and aluminium separately, in the case of zinc−aluminium mixtures, both of them were significantly oxidized. The impurities present on their surface were also oxidized. The type and amount of phases formed during cementation depends on the temperature as well as on the total redox potential of the solution or individual metal pairs, the content and concentration of dissolved impurities in the carbonate solution, etc.

Zinc oxide with a purity of almost 97% can be obtained by subsequent treatment of the leachates via crystallization at 105 °C and calcination at 900 °C for 4 h. The remaining fraction consists of phases such as dicalcium ferrite, willemite and calcium oxides. Elements such as silicon and calcium are contaminants which are removed by the cementation only partially, but their minimal presence does not affect the quality of the product. ZnO with the achieved purity can be used in the rubber industry or in the production of semiconductor components called varistors.

## Figures and Tables

**Table 1:** Predicted chemical reactions of cementation of accompanying metals from ammonium carbonate leachate using zinc at 20 and 80 °C.

Chemical Reaction	ΔG^0^ [kJ/mol]		N°
	20 °C	80 °C	
Cu^+2^ + 1.5Zn + 3 CO_3_^−2^ + 3H_2_O = 1.5Zn(OH)_2_ + Cu + 3HCO_3_^−^ + e^−^	−362.18	−395.26	(1)
Cu^+2^ + Zn + 4CO_3_^−2^ +4NH_4_^+^ = [Zn(NH_3_)_4_]^+2^ + Cu + 4HCO_3_^−^	−282.44	−318.09	(2)
Pb^+2^ + 1.5Zn + 3CO_3_^−2^ + 3H_2_O = 1.5Zn(OH)_2_ + Pb + 3HCO_3_^−^ + e^−^	−273.33	−301.53	(3)
Fe^+2^ + 1.5Zn + 3CO_3_^−2^ + 3H_2_O = 1.5Zn(OH)_2_ + Fe + 3HCO_3_^−^ + e^−^	−218.10	−253.33	(4)
Pb^+2^ + Zn + 4CO_3_^−2^ + 4NH_4_^+^ = [Zn(NH_3_)_4_]^+2^ + Pb + 4HCO_3_^−^	−193.58	−224.34	(5)
2Pb^+2^ + Zn + 4CO_3_^−2^ + H_2_O = ZnO + 2PbCO_3_ + 2HCO_3_^−^ + 2e^−^	−171.20	−195.41	(6)
Cr^+3^ + 1.5Zn + 6CO_3_^−2^ + 6NH_4_^+^ = 1.5[Zn(NH_3_)_4_]^+2^ + Cr + 6HCO_3_^−^	−118.94	−181.68	(7)
Fe^+2^ + Zn + 4CO_3_^−2^ + 4NH_4_^+^ = [Zn(NH_3_)_4_]^+2^ + Fe + 4HCO_3_^−^	−125.86	−161.75	(8)
2Fe^+2^ + Zn + 4CO_3_^−2^+ H_2_O = ZnO + 2FeCO_3_ + 2HCO_3_^−^ + 2e^−^	−157.27	−188.45	(9)
2Cu^+2^ + Zn + 4CO_3_^−2^+ H_2_O = ZnO + 2CuCO_3_ + 2HCO_3_^−^ + 2e^−^	−153.81	−182.21	(10)
Cr^+3^ + 1.5Zn + 3CO_3_^−2^+ 3H_2_O = 1.5(ZnOH)_2_ + Cr + 3HCO_3_^−^	−98.13	−140.49	(11)

**Table 2:** Predicted chemical reactions of cementation of accompanying metals from ammonium carbonate leachate using aluminium at 20 and 80 °C.

Chemical Reaction	ΔG^0^ [kJ/mol]		N°
	20 °C	80 °C	
Cu^+2^ + Al + 3CO_3_^−2^ + 3H_2_O = Al(OH)_3_ + Cu + 3HCO_3_^−^ + e^−^	−670.50	−703.65	(12)
Pb^+2^ + Al + 3CO_3_^−2^ + 3H_2_O = Al(OH)_3_ + Pb + 3HCO_3_^−^ + e^−^	−578.00	−604.32	(13)
Fe^+2^ + Al + 3CO_3_^−2^ + 3H_2_O = Al(OH)_3_ + Fe + 3HCO_3_^−^ + e^−^	−526.41	−561.71	(14)
Cr^+3^ + Al + 3CO_3_^−2^ + 3H_2_O = Al(OH)_3_+ Cr + 3HCO_3_^−^	−394.66	−435.15	(15)
6Fe^+2^ + 2Al + 12CO_3_^−2^ + 3H_2_O = Al_2_O_3_ + 6FeCO_3_ + 6HCO_3_^−^ + 6e^−^	−261.91	−292.99	(16)
6Pb^+2^ + 2Al + 12CO_3_^−2^ + 3H_2_O = Al_2_O_3_ + 6PbCO_3_ + 6HCO_3_^−^ + 6e^−^	−275.85	−299.94	(17)
6Cu^+2^ + 2Al + 12CO_3_^−2^ + 3H_2_O = Al_2_O_3_ + 6CuCO_3_ + 6HCO_3_^−^ + 6e^−^	−258.27	−285.73	(18)

**Table 3:** Chemical composition of input dusts.

(wt.%)	Zn	Fe	Pb	Cd	Cu	Cr	Mn	Ni	Ca	Si	Mg	Cl^−^
**Input EAFD**	28.01	26.34	0.73	0.02	0.12	0.31	1.90	0.013	5.25	1.44	1.94	1.77
**Washed**	28.06	27.17	0.70	0.02	0.11	0.16	1.79	0.013	3.66	1.44	1.83	1.48

**Table 4:** Tap water analysis [39].

Analysed Parameter	Total Hardness	NO_3_^−^	Fe	Mn	Free Chlorides	pH
	(mmol/L)	(mg/L)	(mg/L)	(mg/L)	(mg/L)	
**Tap water**	1.30	3.50	0.080	<0.030	<0.10	8.0
**Limit**	1.1–5.0	50.0	0.20	0.05	0.30	6.50–9.50

**Table 5:** Chemical composition of leachates used for cementation.

Leachate N°	Zn	Fe	Pb	Cd	Cu	Cr	Mn	Ca	Cl^−^	SO_4_^2−^	pH
	(µg/mL)								(g/L)		
1	4552	7.642	0.464	0	0	0.052	0.494	44.952	5.32	-	8.28
2	8250	4.460	21.39	0	7.590	1.320	1.430	57.200	3.90	0.57	9.02
3	11,750	2.670	1.510	0	9.310	4.100	0.945	88.800	2.98	0.62	8.89

**Table 6:** Results of cementation with pure Zn 5 g/L, 20 and 60 °C, 30 min.

N°	(°C)	Leachate 3	Zn	Fe	Pb	Cu	Cr	Mn	Ca	pH
			(µg/mL)							
1	20	Before cementation	11,750	2.67	1.51	9.31	4.10	0.95	88.8	8.82
		After cementation	12,100	2.56	0.78	0.30	0	0	28.4	8.81
		**ƞ** **(%)**	**-**	**2.99**	**48.34**	**96.78**	**100**	**100**	**68.01**	
2	60	Before cementation	11,750	2.67	1.51	9.31	4.10	0.95	88.8	8.82
		After cementation	12,460	0	0.46	0.41	0	0	26.9	8.9
		**ƞ** **(%)**	**-**	**100**	**69.54**	**95.49**	**100**	**100**	**69.71**	

**Table 7:** Results of cementation with pure Al 5 g/L, 20 and 60 °C, 30 min.

N°	(°C)	Leachate 3	Zn	Fe	Pb	Cu	Cr	Mn	Ca	pH
			(µg/mL)							
3	20	Before cementation	11,750	2.67	1.51	9.31	4.10	0.95	88.8	8.82
		After cementation	11,500	2.02	0.55	1.63	0	0	28.6	8.82
		**ƞ [** **%** **]**	**-**	**24.35**	**63.56**	**82.49**	**100**	**100**	**67.79**	
4	60	Before cementation	11,750	2.67	1.51	9.31	4.10	0.95	88.8	8.82
		After cementation	11,140	1.04	0.48	0.88	0	0	28.0	8.86
		**ƞ [** **%** **]**	**-**	**61.05**	**68.21**	**90.55**	**100**	**100**	**68.47**	

**Table 8:** Results of cementation with Zn:Al = 2.5:2.5 g/L and 5:5 g/L at 20, 40, 60 and 80 °C.

N°	t (°C)	Leachate 2 and 3		Zn	Fe	Pb	Cu	Cr	Mn	Ca	pH
		Cementation Agent	Cementation	(µg/mL)							
5	20 °C	2.5 g/L Zn 2.5 g/L Al	Before	8250	5.54	21.39	7.59	1.32	1.43	57.2	9.02
			After	8180	3.39	0	0	0.62	0	51	9.01
			**ƞ [** **%** **]**	**-**	**38.8**	**100**	**100**	**53**	**100**	**10.8**	**-**
6	20 °C	5 g/L Zn 5 g/L Al	Before	8250	5.54	21.39	7.59	1.32	1.43	57.2	9.02
			After	9000	2.76	0	0	0.64	0	54	9.02
			**ƞ [** **%** **]**	**-**	**50.2**	**100**	**100**	**52**	**100**	**5.6**	**-**
7	40 °C	2.5 g/L Zn 2.5 g/L Al	Before	11,750	2.67	1.51	9.31	4.1	0.945	88.8	8.89
			After	11,180	0.71	0	0	3.25	0	77.4	8.89
			**ƞ [** **%** **]**	**-**	**71**	**100**	**100**	**26**	**100**	**13**	**-**
8	40 °C	5 g/L Zn 5 g/L Al	Before	11,750	2.67	1.51	9.31	4.1	0.945	88.8	8.89
			After	10,560	0	0	0	2.07	0	86.6	8.87
			**ƞ [** **%** **]**	**-**	**100**	**100**	**100**	**49.5**	**100**	**2.5**	**-**
9	60 °C	2.5 g/L Zn 2.5 g/L Al	Before	8250	5.54	21.39	7.59	1.32	1.43	57.2	9.02
			After	9500	1.13	0	0	0.68	0	48	9.00
			**ƞ [** **%** **]**	**-**	**79.6**	**100**	**100**	**49**	**100**	**16.1**	**-**
10	60 °C	5 g/L Zn 5 g/L Al	Before	8250	5.54	21.39	7.59	1.32	1.43	57.2	9.02
			After	9640	0	0	0	0.57	0	49	9.01
			**ƞ [** **%** **]**	**-**	**100**	**100**	**100**	**57**	**100**	**14.34**	**-**
11	80 °C	2.5 g/L Zn 2.5 g/L Al	Before	11,750	2.67	1.51	9.31	4.1	0.945	88.8	8.89
			After	10,600	0	0	0	3.19	0	68.6	8.80
			**ƞ [** **%** **]**	**-**	**100**	**100**	**100**	**22**	**100**	**23**	**-**
12	80 °C	5 g/L Zn 5 g/L Al	Before	11,750	2.67	1.51	9.31	4.1	0.945	88.8	8.89
			After	11,700	0	0	0	3.58	0	65.0	8.88
			**ƞ [** **%** **]**	**-**	**100**	**100**	**100**	**13**	**100**	**27**	**-**

**Table 9:** Chemical composition of obtained calcinates from experiments N° 1–4.

N°	Zn in Leachate (µg/mL)	Input for Calcination (g)	Calcinates (g)	Content (wt.%)				Purity of ZnO (wt.%)
				Zn	Fe	Ca	Si	
1	12,100	2.0	1.27	73.13	0.08	0.25	0	91.02
2	12,460	2.0	1.26	66.51	0.06	0.16	0	82.78
3	11,500	2.0	1.23	62.74	0.08	0.17	0	78.1
4	11,140	2.0	1.24	49.10	0.06	0.14	0	61.1

**Table 10:** Chemical composition of obtained calcinates from experiments N° 5–12.

N°	Zn in Leachate (µg/mL)	Input for Calcination (g)	Calcinates (g)	Content (wt.%)				Purity of ZnO (wt.%)
				Zn	Fe	Ca	Si	
5	8180	4.30	2.60	75.67	0.05	0.31	0.46	94.31
6	9000	3.00	1.86	76.33	0.04	0.26	0.37	95.00
7	11,180	4.68	2.94	75.36	0.06	0.41	0.55	93.80
8	10,560	5.60	3.79	77.61	0.04	0.37	0.46	96.60
9	9640	2.80	1.28	77.21	0.00	0.27	0.45	96.10
10	9500	3.00	1.80	77.67	0.04	0.27	0.44	96.67
11	10,600	5.82	3.74	76.35	0.03	0.40	0.61	95.19
12	11,700	3.64	2.44	74.47	0.03	0.50	0.62	93.03

## Data Availability

Not applicable.
